# An investigation into the ameliorating effect of black soybean extract on learning and memory impairment with assessment of neuroprotective effects

**DOI:** 10.1186/1472-6882-14-482

**Published:** 2014-12-13

**Authors:** Ji Hee Jeong, Hyeon Ju Kim, Seon Kyeong Park, Dong Eun Jin, O-Jun Kwon, Hyun-Jin Kim, Ho Jin Heo

**Affiliations:** Division of Applied Life Science, Institute of Agriculture and Life Science, Gyeongsang National University, Jinju, 660-701 South Korea; Daegyeong Institute for Regional Program Evaluation, Regional Industry, Evaluation Agency for Gyeongbuk, Gyeongsan, 712-210 South Korea

**Keywords:** Amyloid beta protein, Black soybean, Cognition, Epicatechin, PC12 cell

## Abstract

**Background:**

The physiological effects of the non-anthocyanin fraction (NAF) in a black soybean seed coat extract on Aβ-induced oxidative stress were investigated to confirm neuroprotection. In addition, we examined the preventive effect of NAF on cognitive defects induced by the intracerebroventricular (ICV) injection of Aβ.

**Methods:**

Levels of cellular oxidative stress were measured using 2′,7′-dichlorofluorescein diacetate (DCF-DA). Neuronal cell viability was investigated by 3-(4,5-dimethylthiazol-2-yl)-2,5-diphenyltetrazolium bromide (MTT) and lactate dehydrogenase (LDH) assay. To investigate in vivo anti-amnesic effects of NAF by using Y-maze and passive avoidance tests, the learning and memory impairment in mice was induced by Aβ. After in vivo assays, acetylcholinesterase (AChE) activity and level of malondialdehyde (MDA) in the mouse brain were determined to confirm the cognitive effect. Individual phenolics of NAF were qualitatively analyzed by using an ultra-performance liquid chromatography (UPLC) Accurate-Mass Quadrupole Time of-Flight (Q-TOF) UPLC/MS.

**Results:**

A NAF showed cell protective effects against oxidative stress-induced cytotoxicity. Intracellular ROS accumulated through Aβ_1–40_ treatment was significantly reduced in comparison to cells only treated with Aβ_1–40_. In MTT and LDH assay, the NAF also presented neuroprotective effects on Aβ_1–40_-treated cytotoxicity. Finally, the administration of this NAF in mice significantly reversed the Aβ_1–40_-induced cognitive defects in in vivo behavioral tests. After behavioral tests, the mice brains were collected in order to examine lipid peroxidation and AChE activity. AChE, preparation was inhibited by NAF in a dose-dependent manner. MDA generation in the brain homogenate of mice treated with the NAF was decreased. Q-TOF UPLC/MS analyses revealed three major phenolics from the non-anthocyanin fraction; epicatechin, procyanidin B1, and procyanidin B2.

**Conclusions:**

The results suggest that the NAF in black soybean seed coat extracts may improve the cytotoxicity of Aβ in PC12 cells, possibly by reducing oxidative stress, and also have an anti-amnesic effect on the in vivo learning and memory deficits caused by Aβ. Q-TOF UPLC/MS analyses showed three major phenolics; (-)-epicatechin, procyanidin B1, and procyanidin B2. Above results suggest that (-)-epicatechins are the major components, and contributors to the anti-amnesic effect of the NAF from black soybean seed coat.

## Background

Black soybeans (*Glycine max* L. Merr.) have been consumed as a medicinal or functional food in Korea, China, and Japan for thousands of years [1]. Recent advances in antioxidant research show that black soybeans possess a strong inhibitory effect against in vitro low density lipoprotein oxidation [2]; stronger 2-diphenyl-1-picryhydrazyl (DPPH) radical scavenging activity; ferric-reducing antioxidant power (FRAP); and oxygen radical absorbance capacity (ORAC) than yellow soybeans [3]. The antioxidant effects of black soybeans are related to the phenolic pigments in their seed coats [2]. Black soybeans are also used as a nutraceutical food for kidney disease, blood circulation, oxidative stress, and counteracting toxicity because of their various physiological effects [4]. In addition, the black hull contains various polyphenols such as anthocyanins, procyanidins, and catechins, and the functional properties of their phenolics have been studied [5]. Anthocyanins from the black soybean seed coat have reportedly inhibited the expression of inflammation-related genes [6] and the H_2_O_2_-induced cell death of rotator cuff tenofibroblasts [7].

Amyloid β peptide (Aβ) is a major component of senile plaques, and considered to have a causal role in the development and progress of the neurodegenerative aspect of Alzheimer’s disease (AD). Although the mechanism of Aβ-induced neurotoxicity remains ambiguous, collected evidence has suggested that the enhanced oxidative stress evoked by Aβ is associated with the pathogenesis of AD [8]. Furthermore, it is well established that the production of excessive reactive oxidative species (ROS) could lead to neuronal apoptosis in neurodegenerative disorders, such as Aβ-induced neuronal apoptosis. Production of neurotoxic Aβ, primarily Aβ_1–40_ (Aβ_40_) and Aβ_1-42_ (Aβ_42_), and their deposition in insoluble plaques are the major neuropathological hallmarks of AD [9]. Aβ_40_ constitutes approximately 90% of the most abundant cleaved form of larger amyloid precursor protein (APP) and exhibits a toxic effect on neurons in the AD brain, although Aβ_42_ is much more prone to aggregation and more toxic to neurons than Aβ_40_ [10]. In addition, the deposition of Aβ_40_ is required for the development of mature amyloid plaques from the initial deposition of Aβ_42_, an early pathological process of AD. Therefore, similar to Aβ_42_, Aβ_40_ is also often used to generate the rodent model of AD [11].

Only a few studies have performed a comparative analysis that quantifies non-anthocyanins extracted from black soybean seed coat [1]. Furthermore, there is little information available on the possible health benefits of non-anthocyanins that have been extracted from the black soybean seed coat on animal cells that have been exposed to cell-damaging oxidative stress. Therefore, non-anthocyanin fractions were used to evaluate their neuroprotective effects against Aβ-induced oxidative stress using rat pheochromocytoma (PC12) cells. The neuron-like PC12 cell is an appropriate in vitro model for the assessment of the neurotoxic effect of Aβ and is widely used to study molecular mechanisms and to develop neuroprotective agents that reduce neurotoxic symptoms [12]. Behavioral in vivo tests were also performed to elucidate the effect of the NAF against Aβ-induced neurodegeneration in mice. Therefore, the present study was undertaken to investigate beneficial effects of NAF on Aβ-induced cognitive dysfunction in neuron like PC 12 cells and mice. Main phenolics of the NAF from black soybean seed coat were qualitatively identified by using Q-TOF UPLC/MS.

## Methods

### Materials

2′,7′-Dichlorofluorescein diacetate (DCF-DA), vitamin C, thiobarbituric acid (TBA), HEPES, sodium bicarbonate, penicillin, amyloid β protein (Aβ_1-40_), 3-(4,5-dimethylthiazol-2-yl)-2,5-diphenyltetrazolium bromide (MTT) assay kit, lactated dehydrogenase (LDH) release assay kit, tacrine and all other chemicals used were purchased from Sigma-Aldrich Chemical Co. (St. Louis, MO, USA). The PC12 cell line (rat pheochromocytoma cells) was obtained from the Korea cell line bank (Seoul, Korea). Roswell Park Memorial Institute (RPMI) 1640 medium was purchased from Lonza Walkersville, Inc. (Walkersville, MD, USA). Fetal bovine serum was obtained from Gibco BRL (Grand Island, NY, USA).

### Extraction of black soybean

Back soybean was purchased from a local market (Jinju, Korea), in September, 2011, and was authenticated by Institute of Agriculture & Life Sciences, Gyeongsang National University. A voucher specimen was deposited at the Herbarium of the Department of Agronomy, Gyeongsang National University. Black soybean was separated into seed coats and dehulled beans by hand. The phenolics in seed coat were extracted from 10 g of seed coats using 70% acetone (200 mL) by the ultrasound-assisted method [13]. Phenolic extraction of black soybean seed coat was done in three replications. A simple fractionation of black soybean seed coat extracts was performed using preconditioned C_18_ Sep-Pak cartridges to separate anthocyanin from non-anthocyanin fractions [14], and then stored -20°C prior to use. Most of the non-anthocyanins were eluted in the ethyl acetate fraction, which was named the NAF in this research.

### Cell culture

The PC12 cell line was derived from a transplantable rat pheochromocytoma [15]. The cells respond reversibly to nerve growth factor (NGF) by induction of the neuronal phenotype. Cells (KCLB 21721, Korea Cell Line Bank, Seoul, Korea) were propagated in RPMI 1640 medium containing 10% fetal bovine serum, 50 units/mL penicillin, and 100 μg/mL streptomycin.

### Measurement of cellular oxidative stress

Levels of cellular oxidative stress were measured by DCF-DA. PC12 cells were pretreated with various concentrations of the NAF (3, 6, 12, and 25 μg/mL) for 48 h. After 48 h, Aβ and NAF group was treated with the 25 μM Aβ for 2 h. Aβ_1-40_ (0.1 mg) was dissolved in distilled water of 1 mL. At the end of the treatment, cells were incubated in the presence of 50 μM DCF-DA for 50 min. After incubation, dichlorofluorescein (DCF) was quantified using a fluorometer (Infinite F200, TECAN, NC, USA) with a 485 nm excitation filter and a 535 nm emission filter. The results were expressed as percent relative to the oxidative stress level of the control cells, which was set to 100% [16].

### Determination of cell viability

MTT reduction assay was determined using the in vitro toxicology assay kit (TOX-1, Sigma-Aldrich chemical Co., St. Louis, MO, USA). Neuronal PC12 cells were plated at a density of 10^4^ cells/well on 96-well plates in 100 μL of RPMI. The cells were pre-incubated with various concentrations of NAF (3, 6, 12, and 25 μg/mL) for 48 h. After 48 h, Aβ and NAF group was treated with the 25 μM Aβ for 3 h. The amount of MTT formazan product was determined by measuring absorbance using a microplate reader (680, Bio-Rad, Tokyo, Japan) at a test wavelength of 570 nm and a reference wavelength of 690 nm [15].

PC12 cells were precipitated by centrifugation at 250 *g* for 4 min at 4°C, 100 μL of the supernatants was transferred into new wells, and lactate dehydrogenase (LDH) was determined using the in vitro toxicology assay kit (TOX-7, Sigma-Aldrich chemical Co., St. Louis, MO, USA). Damage of neuron like PC12 cell membrane was evaluated by measuring the amount of the intra-cellular enzyme LDH released into the medium [16].

### Animals and in vivo experimental design

Institute of Cancer Research (ICR) mice (male, 4 weeks old) were obtained from Samtako (Osan, Korea). The mice were housed two per cage in a room maintained with a 12 h light-dark cycle, 55% humidity and 24 ± 1°C temperature. All animal procedures were approved by the Institutional Animal Care and Use Committee (IACUC) of Gyeongsang National University (certificate: GNU-120409-M0009), and performed in accordance with the Policy of the Ethical Committee of Ministry of Health and Welfare, Korea. Freeze-dried non-anthocyanin fraction from black soybean seed coat extract were administered orally (5, 10, and 20 mg/kg of body weight) once a day for 3 weeks. After 3 weeks, Aβ_1-40_ was administered via intracerebroventricular (ICV) injection without control group. Negative control group was injected with Aβ_1-40_ only. Aβ was dissolved in 0.85% sodium chloride solution (v/v) and injected intracerebroventricularly into each mouse with a 25 μL Hamilton microsyringe fitted with a 26-gauge needle that was inserted to a depth of 2.5 mm. The injection volume was 5 μL (dose 410 pmol/mouse) [16].

### Y-maze test

The Y-maze test was performed 2 days after the Aβ injection. The maze was made of black-painted plastic, and each arm of the maze was 33 cm long, 15 cm high and 10 cm wide, and was positioned at a constant angle. Each mouse was placed at the end of one arm, and allowed to move freely through the maze during an 8 min period. The sequence of arm entries was recorded manually. A spontaneous alternation behavior was defined as entry into all three arms on consecutive choices in overlapping triplet sets. The percentage spontaneous alternation behavior was calculated as the ratio of actual to possible alternations (defined as the total number of arm entries minus two), multiplied by 100 [16].

### Passive avoidance test

A shuttle box was divided into two chambers, one illuminated and one dark, and separated by a guillotine door. During the training trial, each mouse was placed in the lighted compartment, and then when the mouse entered the dark compartment the door was closed, and the mouse received an inescapable electric shock (0.5 mA, 1 s). The test trial was given 1 day after the training trial, and again the mouse was placed in the lighted compartment and the latency time to enter the dark compartment was measured (the step-through latency maximum testing limit was 300 s) [16].

### Preparation of tissue samples, and determination of ex vivo MDA level and AChE activity

For biochemical studies, mice were sacrificed. Brains were dissected and kept at -70°C before use. Whole brains were homogenized with 10 vol. of cold phosphate buffered saline (PBS) in an ice bath. Homogenates were directly centrifuged 10,000 g for 60 s to obtain the supernatant. Supernatant aliquots were used to determine brain malondialdehyde (MDA) levels, AChE activity and protein content.

The AChE assay was performed according to the colorimetric method of Ellmans [17] using acetylthiocholine iodide as a substrate. Whole brains homogenate (5 μL) was mixed with 65 μL of sodium phosphate buffer (50 mM, pH 8.0) and the mixtures incubated at 37°C for 15 min. Absorbance at 405 nm was read immediately after adding an Ellmans’s reaction mixture [70 μL; 0.5 mM acetylthiocholine and 1 mM 5,5′-dithio-bis(2-nitrobenzoic acid)] in a 50 mM sodium phosphate buffer (pH 8.0) to the above reaction mixture. The results were expressed as percent relative to the activity of the control group (100%).

The MDA level was assayed for products of lipid peroxidation by monitoring thiobarbituric acid reactive substance formation. In brief, 160 μL of each homogenate was mixed with 960 μL of phosphoric acid (1%, v/v) followed by addition of 320 μL thiobarbituric acid solution (0.67%, w/w). The mixture was incubated at 95°C in a water bath for 1 h. After cooling, the colored complex was separated by centrifugation (1,600 g for 10 min), and absorbance was measured at 532 nm using tetramethoxypropane as a standard. MDA levels were expressed as nanomole per milligram of protein [16].

### Identification of main phenolics with Q-TOF UPLC/MS

Individual main phenolics in the NAF from black soybean seed coat were qualitatively analyzed by using an ultra-performance liquid chromatography (UPLC) Accurate-Mass Quadrupole Time of-Flight (Q-TOF) UPLC/MS (Agilent Technologies, Santa Clara, CA, USA). That was operated with an electrospray source in negative ion mode to obtain MS and MS/MS data. Separation of phenolics was performed on a ACQUITY UPLC® BEH C_18_ column (2.l × 100 mm, 1.7 μm particle size; Waters Corp, Milford, MA, USA) with a flow rate of 0.3 ml/min, and oven temperature of 40°C. A linear solvent gradient of binary mobile phase (solvent A, 0.1% formic acid in distilled water; solvent B, 0.1% formic acid in acetonitrile) during analysis was applied as follows: 99% A/1% B at 0 min, 99% A/1% B at 2 min, 50% A/50% B at 8 min, 50% A/50% B at 10 min, and 99% A/1% B at 12 min. The conditions for MS analyses included the drying gas (N_2_) temperature at 350°C, drying gas flow at 10 l/min, nebulizer pressure at 45 psi, fragmentor voltage at 175 V, and capillary voltage at 4000 V. Mass range was set from m/z 100 to 1000.

### Statistical analysis

All data were expressed as mean ± SD. Each experimental set was compared by one-way analysis of variance (ANOVA) and Duncan’s multiple-range test (*P* < 0.05) using SAS software (SAS Institute Inc., Cary, NC, USA).

## Results and discussion

### Measurement of cellular oxidative stress

Oxidative stress caused by increased accumulation of ROS has been implicated in neurodegenerative diseases such as AD [16]. Oxidative stress in AD may result from aging, energy deficiency, inflammation, or excessive production of Aβ. Aβ can induce cell death through a mechanism involving hydrogen peroxide etc. [18, 19].

**Figure 1:**
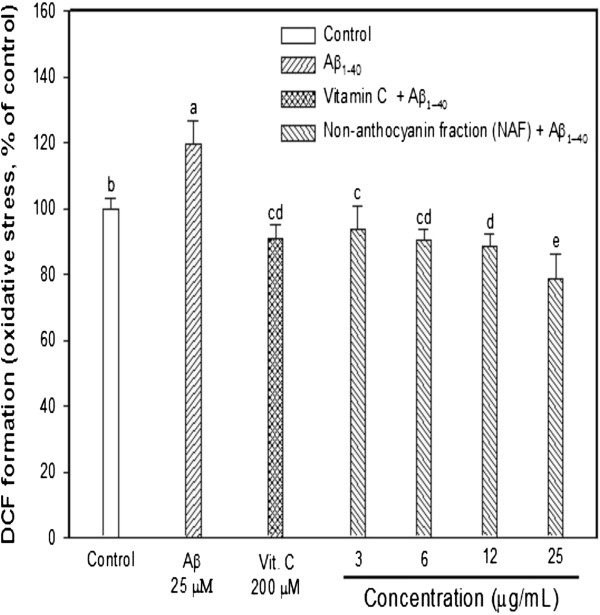
**Effect of the NAF from black soybean seed coat on ROS production determined in the presence and absence of Aβ in PC12 cells.** Results were expressed as mean ± SD in triplicate. Significant difference (*p <* 0.05 versus vitamin C) was observed on the Aβ-induced cell death. Different superscripts indicate significant difference among groups at *p* < 0.05.

The decreased oxidative stress values were measured using a DCF-DA assay [16]. Intracellular ROS accumulation resulting from Aβ treatment was significantly reduced when cells were treated with NAF when compared with those only treated with Aβ (Figure 1). At the level of 200 μM vitamin C as a positive control, decreased oxidative stress to about 29% below that of the only-Aβ treatment group. The NAF group (3-25 μg/mL) resulted in a 94-79% decrease of cellular ROS levels compared to the Aβ exposure. These results suggest that the NAF group protected PC12 cells from oxidative toxicity.

### Cytoprotective effects of the non-anthocyanin fraction

**Figure 2:**
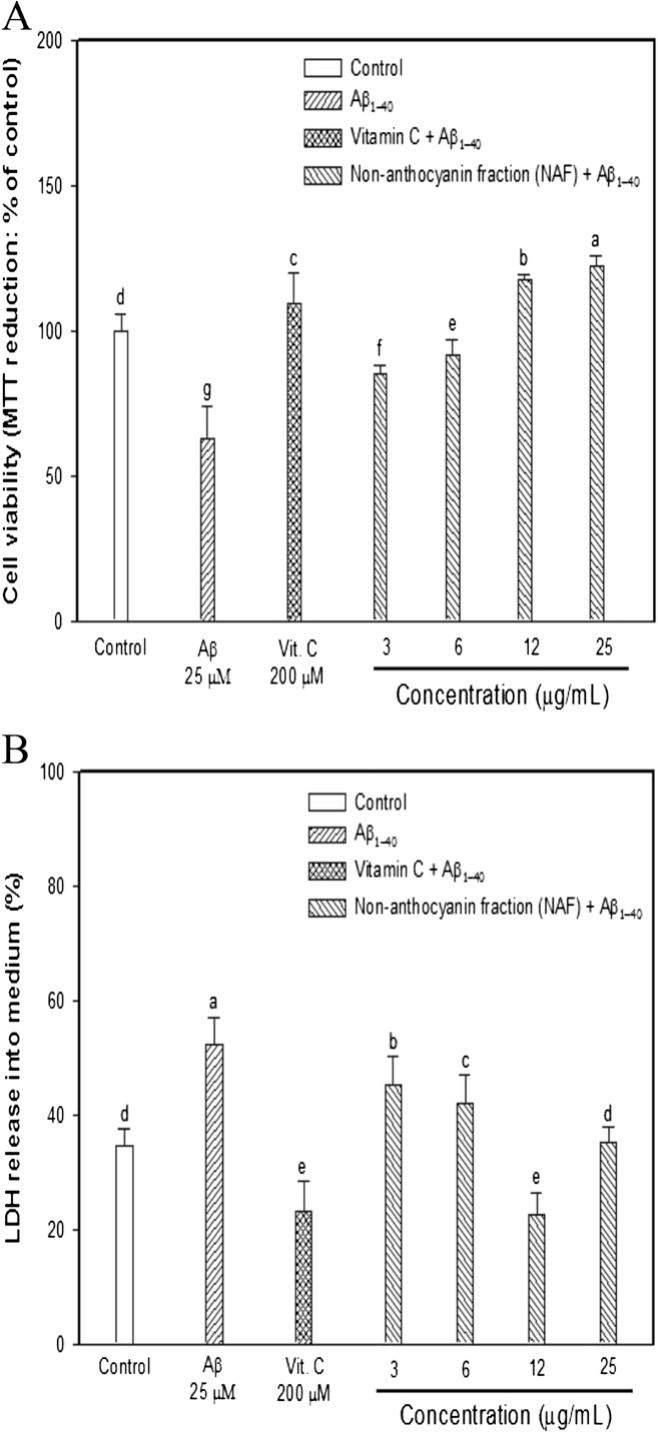
**Cytoprotective effect of the NAF from black soybean seed coat on Aβ-induced neurotoxicity in PC12 cells. (A)** Protective effect of the NAF on Aβ-induced cytotoxicity in PC12 cell system. PC12 cells were pretreated for 48 h with various concentrations. After 48 h, the cells were treated with 25 μM Aβ for 3 h. **(B)** Inhibition of lactate dehydrogenase release of the NAF on Aβ-induced membrane injury in PC12 cell. Bars were represented with the mean ± SD (n = 3). Significant difference (*p <* 0.05 versus vitamin C) was observed on the Aβ-induced cell death. Different superscripts indicate significant difference among groups at *p* < 0.05.

Deposits of Aβ and neurofibrillary tangles are the two pathological hallmarks of AD. There is recent evidence that Aβ aggregates can impair the functionality, morphology, and the subsequent viability of neuronal cells [19]. Aβ, in the form of insoluble fibril deposits, is an important constituent of senile plaques in AD patients, and it has been suggested to be the cause of the neurodegeneration that occurs in AD brains [20]. To evaluate Aβ neurotoxicity properly, it is important to use an appropriate method for monitoring cell viability. Consequently, an MTT reduction assay was used to investigate Aβ-induced neurotoxicity. The MTT dye reduction assay is based on the catalytic activity of some metabolic enzymes in entire mitochondria, and mitochondria may be one of the most sensitive primary targets of oxidative injury in neuronal cells [21]. Aβ caused a decrease in cell viability (about 63%), but pretreatment cells with increasing concentrations of NAF inhibited Aβ-induced neurotoxicity (Figure 2A). The viability of cells with the NAF was about 86–123% at concentrations ranging from 3 to 25 μg/mL. In particular, the group with ≥12 μg/mL displayed more effective protection than those in which vitamin C was used as a positive control. The results of this study suggest that the neuron-like PC12 cell protection by NAF derived from black soybeans might be due to the mitochondrial protection.

Another assay in PC12 cell membranes was performed to confirm the neuroprotective effect of the non-anthocyanin fraction. An LDH assay is a means of measuring either the number of cells via total cytoplasmic LDH or membrane integrity as a function of amount of cytoplasmic LDH released into the medium [22]. Lipid peroxidation is increased in neurodegenerative diseases such as AD. Polyunsaturated fatty acid levels, especially arachidonic acid and docosahexaenoic acid, are high in the neuronal cells of the brain. They are more vulnerable to attack by ROS, peroxidation of which can lead to changes in membrane integrity and fluidity [15]. Because the neuronal plasma membrane is sensitive to oxidative stress, the cell membrane protective effect of the NAF derived from black soybean seed coat extracts on Aβ-induced neurotoxicity was investigated by the LDH release assay, measuring the activity of this stable enzyme released into the medium from apoptotic PC 12 cells. In this LDH assay, Aβ acted as an oxidative inducer that increased the plasma membrane damage in PC12 cells, whereas the NAF efficaciously protected against membrane loss. In other words, as shown in Figure 2B, Aβ treatment caused an increase in LDH release into the medium (52%), but the NAF treatment efficiently inhibited cytoplasmic LDH enzyme release. These results presented that the NAF could protect PC12 cells against the lesions induced by Aβ-induced neurotoxicity. A statistical correlation between in vitro antioxidant and neuroprotective effects was examined. The first order of regression analysis between DCF-DA assay and MTT assay had a correlation coefficient (*r*^*2*^) of 0.696. Finally, the data suggested that the PC12 cell protection effect of the NAF is partially due to the mitochondrial and cell membrane protection effect working against the Aβ-induced neurotoxicity.

### Effect of the NAF on Y-maze and passive avoidance test

**Figure 3:**
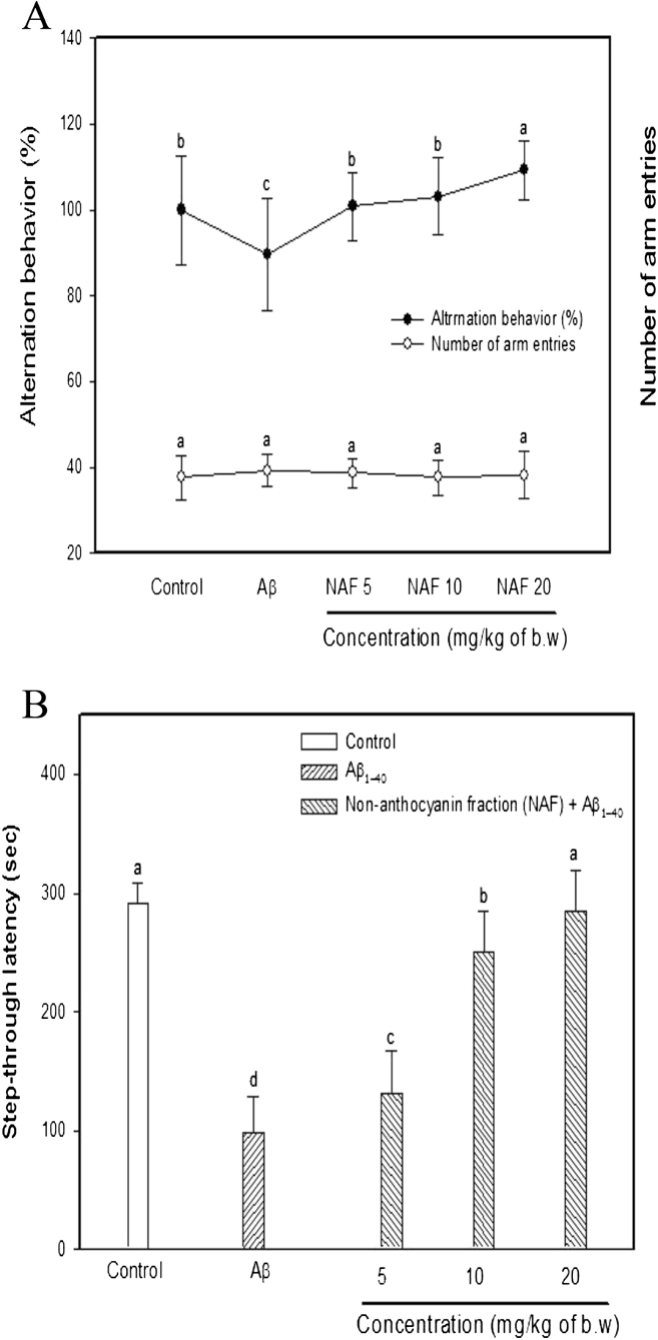
**Effect of the NAF from black soybean seed coat on Y-maze (A) and passive avoidance test (B).** Control group was injected with saline (0.85%). Aβ_1-40_ was injected with 410 pmol of Aβ_1-40_ per mouse. Sample groups were injected Aβ_1-40_ followed by feeding with the NAF (5, 10 and 20 mg/kg per day, respectively). Bar graphs were represented with the mean ± SD (n = 8) (*p <* 0.05 versus control group, *p <* 0.05 versus Aβ group). Different superscripts indicate significant difference among groups at *p* < 0.05.

The effect of dietary administration of NAF on behavioral abilities was examined using the animal model based on an intracerebroventricular (ICV) Aβ injection [10, 16]. Cognitive effects were evaluated both in a Y-maze and by using a passive avoidance test. The Y-maze is a hippocampal-dependent test that evaluates long-term spatial memory. This test is different from the passive avoidance test since the Y-maze is based on the subject’s innate inclination to explore a new environment, rather than on learning a new behavior or rule [23]. Exposure to Aβ causes impairments in the learning and memory systems of mice. It has also been reported that antioxidants can protect neuronal cells against Aβ-induced cytotoxicity [16]. In Figure 3A, the mice injected with Aβ_1-40_ exhibited significantly impaired spatial working memories (10.50% decrease in alternation behavior) compared with that of the control group (100%). The groups that were pretreated with the sample increased their spontaneous alternation behavior after Aβ injection (the non-anthocyanin fraction 5 mg/kg: N5, 100.83%; 10 mg/kg: N10, 103.12%; 20 mg/kg: N20, 109.24%). In contrast, the number of arm entries did not change between all the experimental groups, which demonstrated that general locomotor-activity was not affected by Aβ_1-40_ (Figure 3A). Our previous research presented that pretreatment with the chestnut inner skin fraction including gallic acid and catechins increased spontaneous alternation behavior in the Aβ-injected mice (5 mg/kg of the sample: 91%; 10 mg/kg of the sample: 98%; 20 mg/kg of the sample: 104%) [24]. These comparing results suggest that a diet containing NAF of black soybean seed coat have slightly higher protective effect against Aβ-induced cognitive loss.

The passive avoidance test is an amygdala-dependent test that evaluates the ability of mice to learn and to retain an associative rule. It had been reported to be related to 'long-term’ or reference memory. N-Methyl-D-aspartic acid (NMDA) receptors are involved in the formation of post-training memory in the amygdala and hippocampus [25]. The passive avoidance paradigm has been used to study learning and memory associated with stressful stimulus. The procedure is based on the innate preference of rodents for the dark compartment of the apparatus and the suppression of this innate preference following exposure to an inescapable shock; that is, passive avoidance performance is an adaptive response to a stressful experience that serves as a measure of learning and memory [26]. As shown in Figure 3B, mice treated with the NAF exhibited attenuated Aβ-induced impairment in a dose-dependent manner. The passive avoidance test was carried out 5 days after Aβ_1-40_ injection. The Aβ-injected mice displayed a significant reduction (66.24% decrease) in step-through latency compared with the control group. The NAF attenuated the Aβ-induced impairment of the mice in the passive avoidance test. A statistical correlation between in vivo experiments was estimated. The first order of regression analysis between Y-maze test and passive avoidance test had a correlation coefficient (*r*^*2*^) of 0.714. Therefore, these results indicate that the NAF displayed a significant anti-amnestic effect in the Aβ-induced mouse model.

### Effect of the NAF on AChE activity and lipid peroxidation level in Aβ-injected mice brain

**Figure 4:**
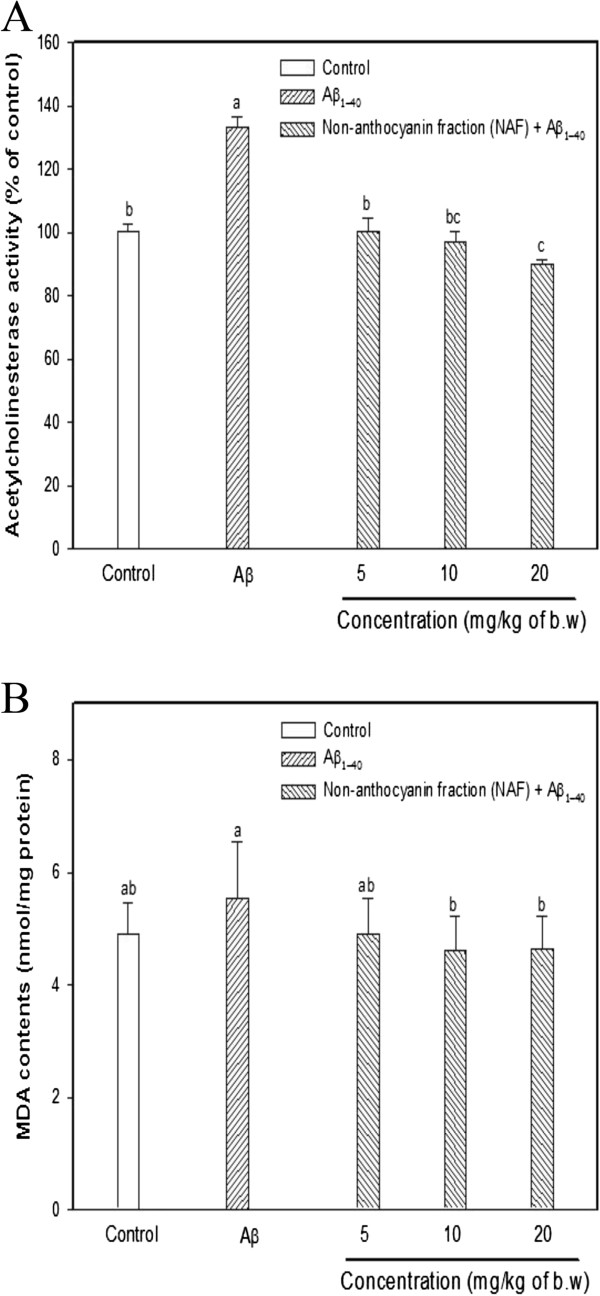
**Effect of the NAF from black soybean seed coat on AChE activity (A) and lipid peroxidation (B) from Aβ-induced mice brain homogenate.** Control group was injected with saline (0.85%). Aβ_1-40_ was injected with 410 pmol of Aβ_1-40_ per mouse. Sample groups (NAF5, NAF10, and NAF20) were injected with Aβ_1-40_ followed by feeding with the NAF (5, 10, 20 mg/kg per day, respectively). Bar graphs were represented with the mean ± SD (n = 8) (*p <* 0.05 versus control group, *p <* 0.05 versus Aβ group). Different superscripts indicate significant difference among groups at *p* < 0.05.

Current studies suggest that one of the fundamental characteristics of learning and memory impairment is the widespread degeneration and dysfunction of the cholinergic system. Aβ injection directly inhibits various cholinergic neuronal functions [10]. The involvement of the cholinergic system in learning/memory has long been established. AChE normally has a strong temporal association with the detection of novel or behaviorally significant stimuli and thus excessive AChE release might impair tasks that require learning novel stimuli [27, 28]. AChE activity has been shown to be increased within and around amyloid plaques, to promote the assembly of amyloid beta-peptides into fibrils, and to increase the cytotoxicity such as oxidative stress of these peptides [29]. In accordance, higher AChE inhibition does not necessarily mean better cognitive performance and the findings denote that there is an optimal balance between cholinergic neurotransmission and cognitive performance. Besides, AChE activity is also found in brain regions with low or no cholinergic inputs, such as the substantia nigra, cerebellum, globus pallidus, and the hypothalamus, where it exerts non-enzymatic neuromodulatory functions that affect neurite outgrowth, synaptogenesis, the modulation of the activity of other proteins, regional cerebral blood flow, and other functions [30, 31]. After in vivo assays, AChE activity in the mouse brain was determined to investigate the physiological effect of the non-anthocyanin fraction. As shown in Figure 4A, the Aβ group without sample treatment showed an increase in AChE activity, while the pretreatment groups with the NAF had relatively inhibited AChE in the brains of the mice. In particular, the AChE activity of the N20 group was significantly decreased when compared with that of the Aβ group.

We also examined whether the NAF could inhibit Aβ-induced lipid peroxidation by measuring the levels of malondialdehyde (MDA), a marker of lipid peroxidation, in the brain homogenates. ROS and the susceptibility of brain tissue to oxidative stress have been well reviewed [32]. In particular, polyunsaturated fatty acids (PUFAs, e.g., arachidonic acid etc.) are highly sensitive to oxidative modification due to their double bonds. Furthermore, the central nervous system is more susceptible to oxidative stress because it consumes approximately 20% of the total respired oxygen, even though it comprises only 2% of the body’s weight. It is also known that the protective system in the brain is poor against oxidative stress, compared to other tissues [33]. Of the manifestations of oxidative damage, we specifically measured the lipid peroxidation end product, as it is one of the most commonly used biomarkers in neurodegeneration research. Several studies have found lipid peroxidation changes and MDA elevations in brain tissue [34]. The levels of MDA were found to have increased (0.6 nmole/mg protein) in the Aβ group when compared with that of the control group. The NAF administration significantly lowered the MDA concentration in a dose-dependent manner (Figure 4B). Therefore, it can be concluded that NAF attenuate the toxicity of Aβ through its action as an antioxidant in the brain tissue.

### Identification and Q-TOF UPLC/MS fingerprint of main phenolics in the NAF of black soybean seed coat

Main phenolics of the NAF from black soybean seed coat were qualitatively identified by the Q-TOF UPLC/MS analysis for retention time, UV-VIS spectrum, MS^2^ scan for mass fragmentation, and the comparison of MS spectrum obtained by Q-TOF LC/MS (MS/MS) to fragmentation data from previous literature reports [35–37].

**Figure 5:**
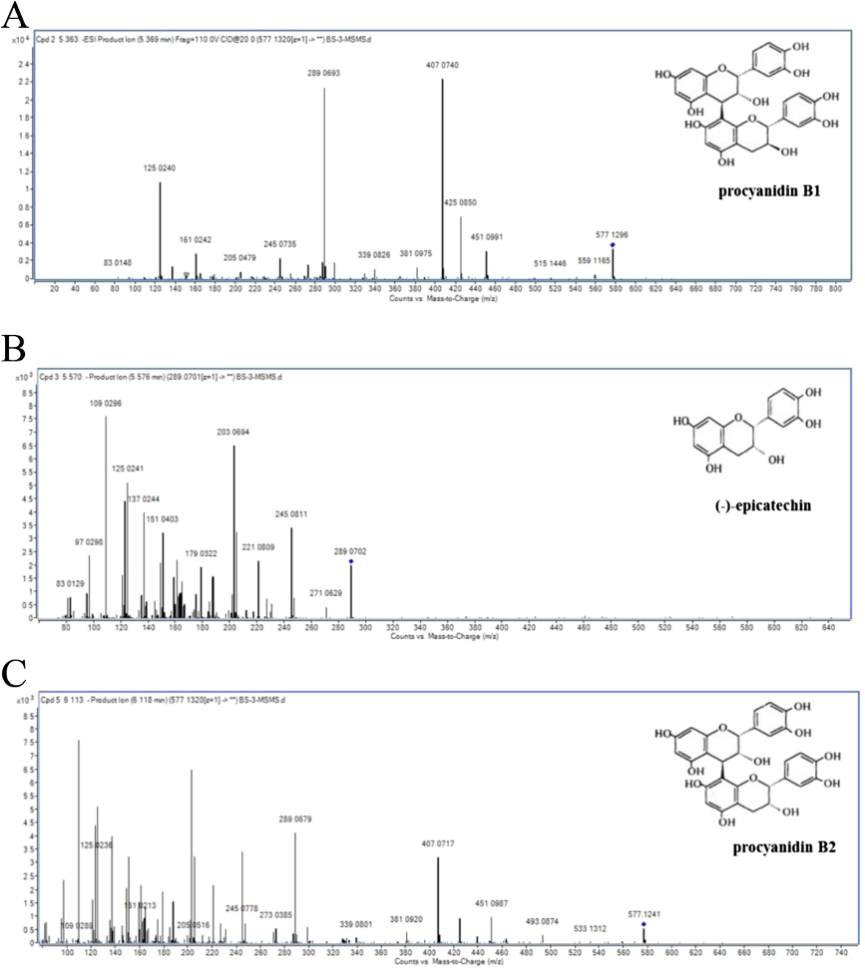
**Q-TOF UPLC/MS spectra in negative ion mode and chemical structures of procaynidin B1 (A), (-)-epicatechin (B), and procyanidin B2 (C).**

Full scan data showed that three phenolics (Compound A, B, and C) exhibited the molecular ions M^-^ m/z values of 577.1296, 289.0702, and 577.1241, respectively (Figure 5). In addition, MS^2^ scan data of compound A produced two major fragments at m/z 425.0850, 407.0740, 289.0693, and 125.0240. Based on the UV-VIS spectrum, retention time, and the comparison of MS spectrum obtained by Q-TOF LC/MS to fragmentation data from previous literature reports [35, 37], compound A was provisionary analyzed as a procyanidin B1. Compound B as a main phenolics exhibited a molecular ion at m/z 289.0702 and fragment ions at m/z 245.0811, 203.0694, 179.0322, 161.0601, 151.0403, 137.0244, 125.0241, and 109.0296. Consequently, compound B was identified as epicatechin, based on the UV-VIS spectrum/retention time of commercial epicatechin standard, and the comparison of MS spectrum obtained by Q-TOF LC/MS to fragmentation data from previous literature reports [36, 37]. Finally, main fragments of compound C containing the structural information for these compounds were those at m/z 407.0717, 289.0679, 273.0385, and 125.0236. Compounds C was tentatively identified as a procyanidin B2, based on the UV-VIS spectrum, retention time, and the comparison of MS spectrum obtained by Q-TOF LC/MS to fragmentation data from previous literature reports [35, 37].

Black soybean has been a functional food for many years in Asia. Recently, many researches have focused on the physiological activities of the seed coats, which are rich in various phenolics, such as (epi)catechins, proanthocyanidins (PA), and other flavonoids [1, 2, 5]. PAs as condensed tannins are present in various foodstuffs, and they are polymers of flavan-3-ols which present a wide variety of chemical structures: among their structural units there may be (epi)catechin, (epi)gallocatechin or (epi)afzelechin units, in which case they are called procyanidins (PC), prodelphinidins, and propelargonidins, respectively. PA can be divided into several sub-classes, of which the PC, exclusively consisting of (epi)catechin units and their galloyl derivatives, is the most abundant. The monomeric units of PC are linked through a C4-C8 or C4-C6 bond (B-type), which can coexist with an additional C2-O-C7 or the less abundant C2-O-C5 bond (A-type) [35, 37]. In our Q-TOF UPLC/MS results, MS^2^ scan data also showed that compounds A, B, and C made the fragmentation of (epi)catechin (m/z 289), indicating that all of them were derivatives of procyanidin B group.

(+)-Catechin and (-)-epicatechin are flavonoids that exist widely in natural products or functional food substances. Catechins in green tea, namely (-)-epicatechin, (-)-epicatechin-3-gallate, (-)-epigallocatechin, (-)-epigallocatechin-3-gallate, (+)-catechin, and (+)-gallocatechin, were found to have antioxidant, anti-cancer, hypocholesterolemic, anti-ageing, and anti-inflammatory effects [38]. The antioxidation mechanism of (+)-catechin is considered to be due to the change of the B-ring to an ο-quinone. (-)-Epicatechin was also confirmed to give a similar result. This changed ο-quinone structure has stronger antioxidative activity [39]. Besides, (epi)catechins have been reported to prevent Aβ-induced oxidative damage in cultured neuronal cells [22]. And several in vitro and in vivo studies suggest that catechins may affect potential targets associated with the progression of neurodegenerative disease such as AD [40, 41]. Aβ_25-35_ is known to be the toxic domain of the Aβ_1-40(42)_, where methionine, at position 35, seems to be responsible for the redox site conformation. It induces severe neuronal damage in several ways, such as the increase of intracellular calcium, oxidative stress, apoptosis, and necrosis that may be responsible for the cognitive deficit [42].

Soybean varieties including black soybeans contain plentiful concentration of physiological phenolics such as anthocyanin sans isoflavones. Among the several anthocyanins, cyanidin-3-glucoside (C-3-G) is the most abundant component, consisting of about 70% of the total anthocyanins in black soybean seed coats [43]. It has been presented that C-3-G as anthocyanincs has strong antioxidant activity, and protective effect against oxidative stress-induced PC12 cell death [44]. Recent research also reported the protective effect of C-3-G which is able to cross the blood-brain-barrier and can localize to brain, against ischemia-reperfusion injury in the brain tissue [45, 46]. In addition, another study reported that soybean isoflavone improved Aβ-induced learning and memory deficit, because soybean isoflavone such as genistein prevented Aβ_1-40_ induced pathological changes through antioxidant pathway in rats [47, 48]. Soybean isoflavone also reverse Aβ-induced down-regulation of neuronal synaptic plasticity such as synaptophysin, PSD-95, CaM, CaMK II, CREB, pCaMK II (Thr286), and pCREB (Ser133) [49]. Consequently, we also found that Aβ increased oxidative stress in PC12 cell, and consequently lipid peroxidation, accompanied by loss of learning and memory deficits, and increased AChE/MDA level in the mice brain tissue. However, (-)-epicatechin, found as main phenolics in the non-anthocyanin fraction, seem to be the major contributors to in vitro neuronal cell protective effect against Aβ-induced oxidative stress, and they attenuate the learning and memory deficits through inhibition of AChE/ MDA formation in the present study. Therefore, our results show a new possibility to search for effective treatments against the neurodegenerative disease such as AD, although more investigations are needed to understand the precise mechanisms of action of these phenolics in the NAF from black soybean seed coat.

## Conclusions

It can be concluded that NAF derived from black soybean seed coat extract inhibit the neuronal cell death caused by Aβ-induced cytotoxicity, and improve Aβ-induced learning and memory deficits. After completing the in vivo behavioral tests, important biochemical markers in the brain tissues of the mice also confirmed the protective effects of the non-anthocyanin fraction. The potential neuroprotective effects of the NAF are linked to inhibition of AChE and MDA production in mice brain tissue with Aβ-induced amnesia. Therefore, NAF including epicatechin as a main contributor might be effective in preventing the onset of learning and memory impairment, and in ameliorating cognitive defects in the early phases of neurodegeneration.

## Abbreviations

DCF-DA: 2′,7′-dichlorofluorescein diacetate
MTT: 3-[4,5-dimethythiazol-2-yl]-2,5-diphenyl tetrazolium bromide
LDH: Lactate dehydrogenase
AChE: Acetylcholinesterase
MDA: Malondialdehyde
DPPH: 2,2-diphenyl-1-picryhydrazyl
FRAP: Ferric-reducing antioxidant power
ORAC: Oxygen radical absorbance capacity
Aβ: Amyloid β peptide
AD: Alzheimer’s disease
ROS: Reactive oxidative species
APP: Amyloid precursor protein
PC12: Pheochromocytoma
TBA: Thiobarbituric acid
RPMI: Roswell Park Memorial Institute
NGF: Nerve growth factor
DCF: Dichlorofluorescein
ICR: Institute of cancer research
ICV: Intracerebroventricular
PBS: Phosphate buffered saline
ANOVA: One-way analysis of variance
NAF: Non-anthocyanin fraction
NMDA: N-Methyl-D-aspartic acid
Q-TOF: Quadrupole time of-flight
PA: Proanthocyanidins
PC: Procyanidins.
